# Evaluation of a Novel Pan-RAS Inhibitor in 3D Bioprinted Tumor Models

**DOI:** 10.3390/cancers17182958

**Published:** 2025-09-10

**Authors:** Daniela D. De Nobrega, Logan C. Eiler, Parmanand Ahirwar, Sonika Nallapu, Urvi P. Rawal, Chelsea L. Crawford, Donald J. Buchsbaum, Adam B. Keeton, Yulia Y. Maxuitenko, Xi Chen, Gary A. Piazza, Allan Tsung, Karim I. Budhwani

**Affiliations:** 1 CerFlux, Birmingham, AL 35203, USAlce@cerflux.com (L.C.E.); sn@cerflux.com (S.N.);; 2 Drug Discovery and Development, Harrison College of Pharmacy, Auburn University, Auburn, AL 36849, USAyym0001@auburn.edu (Y.Y.M.);; 3 Department of Surgery, School of Medicine, University of Virginia, Charlottesville, VA 22901, USA

**Keywords:** new approach methods, drug discovery, 3D bioprinting, colorectal cancer, RAS mutations, pan-RAS inhibitors, ex vivo tumor models

## Abstract

**Background**: Colorectal cancer (CRC) remains a significant global health burden, with KRAS mutations driving ~40% of cases. The efficacy of recently approved, mutant-specific KRAS inhibitors is limited by mutational status as well as intrinsic and adaptive resistance mechanisms. Pan-RAS inhibitors, such as ADT-007, offer broader therapeutic potential by targeting multiple RAS isoforms. Here, we evaluate ADT-007 in 3D bioprinted ex vivo slice tissue (BEST) generated from KRAS-mutant and RAS wild-type (WT) CRC cell lines. **Methods**: Potency and selectivity of ADT-007 were benchmarked against bortezomib (proteasome inhibitor) and YM155 (survivin inhibitor) using high-content imaging and ATP-based luminescence assays. Apoptosis induction was assessed with Annexin V/propidium iodide and flow cytometry. **Results**: ADT-007 exhibited high potency and selectivity in KRAS-mutant BEST, reducing tumor burdens >30% at nanomolar concentrations, and demonstrated superior selectivity with minimal cytotoxicity in WT RAS BEST. Annexin V staining confirmed selective induction of apoptosis in KRAS-mutant cells. **Conclusions**: The selective potency and specificity of ADT-007 warrant further investigation of pan-RAS inhibitors for treating RAS-driven cancers. This study also underscores the translational utility of 3D BEST models for preclinical drug response assessment. Further validation in patient-derived BEST is necessary to evaluate the potential of ADT-007 in clinical settings.

## Simple Summary

Bioprinted 3D tumor models are an innovative approach that replicates the structure and environment of real tumors, offering an alternative to animal models for testing new drugs. In this study, we employ these models to evaluate a novel inhibitor targeting RAS proteins, common drivers of many cancers. By recreating the complex architecture of tumors in the laboratory, we demonstrate that this compound selectively eliminates tumor cells harboring RAS mutations while sparing cells without these mutations. Our work highlights the promise of 3D bioprinted tumor models for guiding drug development and advancing treatment strategies for cancers driven by RAS alterations.

## 1. Introduction

Colorectal cancer (CRC) remains one of the most common and lethal cancers in the world, with an estimated 1.9 million new cases and nearly 1 million deaths in 2022 [1]. Although CRC is more common in high-income countries, recent studies point to increasing incidence in low- and middle-income countries [2]. Despite recent breakthroughs in cancer research and translational medicine, the prognosis for patients with advanced-stage CRC remains poor, particularly for patients harboring KRAS mutations [3,4]. The RAS family of membrane-bound small guanine nucleotide-binding proteins plays a crucial role in transducing signals from extracellular growth factors intracellularly to regulate the proliferation, survival, and differentiation of both normal and cancerous cells [5,6]. Mutations in RAS result in the constitutive activation of RAS isozymes, KRAS, HRAS, or NRAS, which in turn activate downstream signaling pathways, such as RAF-MEK-ERK and PI3K-AKT-mTOR, that promote malignant transformation, disease progression, and metastasis [7,8]. KRAS mutations are responsible for approximately 25% of all human cancers and about 40% of CRC cases [9,10]. Multiple KRAS mutations occur in CRC, including G12D (34%), G12V (21%), G13D (20%), G12C (8%), and others (18%) [11]. Given the critical role of KRAS in oncogenesis, the development of new drugs targeting KRAS has been a focal point of hundreds of cancer research labs around the world, due to their potential for the selective killing of cancer cells with mutant KRAS. However, KRAS was long considered undruggable [12] due to its high affinity for GTP/GDP and the lack of deep pockets for small molecule binding other than the nucleotide-binding domain [13]. The challenges in directly targeting RAS spurred the development of alternative strategies that selectively induce apoptosis, such as inhibiting downstream signaling pathways, although none have significantly improved patient survival [14].

Meanwhile, researchers continue to investigate other therapeutic approaches for treating CRC, including proteasome inhibitors such as bortezomib, which disrupt protein homeostasis and induce apoptosis [15]. The proteasome, a multi-catalytic proteinase complex, is responsible for degrading ubiquitinated proteins and regulating various cellular processes, including cell cycle progression and survival. The ubiquitin–proteasome proteolytic pathway is implicated in regulating key proteins involved in cell cycle progression and major transcription factors such as p53, nuclear factor-κB (NFκB), and hypoxia-inducible factor-1 (HIF-1) [16]. Inhibition of the proteasome disrupts these processes, leading to the accumulation of pro-apoptotic factors and induction of cell death. Bortezomib, [(1R)-3-methyl-1-[[(2S)-1-oxo-3-phenyl-2[(pyrazinylcarbonyl)amino]propyl]amino]butyl] boronic acid, specifically and selectively inhibits the 26S proteasome, comprising a 20S core and a 19S regulatory complex. By stabilizing the inhibitory molecule IκB, proteasome inhibitors induce apoptosis to suppress cancer progression and metastasis [15]. Bortezomib has demonstrated significant antitumor activity in preclinical studies across a wide range of cancers, including CRC [16,17]. Clinical trials have further investigated bortezomib in patients with metastatic CRC and demonstrated modest antitumor activity. These studies examined both clinical outcomes and molecular mechanisms through tumor biopsy analysis, revealing differential effects on HIF-1α and its transcriptional target, carbonic anhydrase IX (CAIX), indicating potential disruption of response to tumor hypoxia following bortezomib treatment [15].

Survivin inhibitors such as YM155 have emerged as another class of apoptosis inducers with potential therapeutic benefits for CRC. Survivin, a member of the inhibitor of apoptosis protein (IAP) family, is highly expressed in most cancers, including CRC, where it plays dual compounding pathogenic roles by promoting cell proliferation and inhibiting apoptosis [18]. Survivin has been implicated in both chemoresistance and mortality among CRC patients [19,20], particularly in CD133+ cancer cells, as demonstrated in studies using the CRC cell line, HT29 [21]. Moreover, the interaction between survivin and CD133 may contribute to disease progression [21,22]. Targeting survivin has thus garnered considerable interest as a therapeutic strategy, with small-molecule inhibitors and antisense oligonucleotides being developed to inhibit survivin function and expression. YM155, 1-(2-Methoxyethyl)-2-methyl-4, 9-dioxo-3-(pyrazin-2-ylmethyl)-4, 9-dihydro-1H-naphtho [2, 3-d] imidazolium bromide, is among the small-molecule survivin inhibitors investigated for its potential to treat CRC [23,24]. However, broad expression of survivin in both cancerous and normal cells raises concerns regarding cancer cell selectivity and associated side effects [25,26].

Although KRAS was historically considered to be undruggable [12], recent advances in technology and drug discovery have made possible the development of KRAS^G12C^ inhibitors, such as sotorasib and adagrasib, which can bind to its inactive GDP-bound state, blocking its activation. Mutant-specific KRAS inhibitors have shown promise not only in preclinical studies but also in clinical trials for non-small cell lung cancer and CRC [27,28]. However, two serious limitations persist due to the allele-specific nature of these inhibitors: (1) the G12C KRAS mutation accounts for only 3% of CRC patients [29], thereby severely limiting clinical utility, and (2) experimental studies have identified multiple resistance mechanisms to allele-specific KRAS inhibitors, including the emergence of new KRAS mutations (e.g., Y96C), KRAS amplification, unchecked activation of co-expressed WT RAS isozymes (e.g., via EGFR stimulation), mutations in NRAS, or acquired bypass mechanisms (MET amplification or new oncogenic fusions) [30,31,32,33,34]. This dual constraint of limited clinical utility and potential for multiple resistance mechanisms underscores the urgent need for pan-RAS inhibitors that target a broader range of RAS mutations that would offer therapeutic benefits to a larger patient population and with reduced potential for resistance.

In recent years, the landscape of KRAS-targeted therapies has evolved significantly with the emergence of alternative targeting strategies and a vast array of novel small-molecule inhibitors. Given the heterogeneity of KRAS mutations that drive CRC, a pan-RAS inhibitor would be expected to have a broader scope of therapeutic use and reach to escape both intrinsic and adaptive mechanisms of resistance. Pan-RAS inhibitors, which target multiple RAS mutations and isoforms simultaneously, have shown promise in preclinical models [35,36]. Despite the long-held belief that pan-RAS inhibitors would be overtly toxic, one such pan-RAS inhibitor, RMC-6236, is currently in Phase III clinical trials [36], while another, ADT-1004, an orally bioavailable prodrug of ADT-007, is in preclinical development [35,37].

Concurrently, advances in additive manufacturing and biomedical engineering [38] have led to the development of innovative tools and technologies that enhance our ability to evaluate these and other new target-directed anticancer agents. For instance, emerging ex vivo cancer models now incorporate more physiologically relevant 3D bioprinted ex vivo slice tissue (BEST) [39,40] instead of traditional 2D monolayer cultures, which are less predictive of clinical efficacy [41,42,43]. BEST constructs have the potential to be more predictive of clinical efficacy by more closely mimicking the 3D tumor microenvironment, allowing for the investigation of drug efficacy in a setting that better recapitulates the complexity of human tumors.

Herein, we evaluate ADT-007, a novel pan-RAS inhibitor, and benchmark its potency and selectivity against other known apoptosis inducers, bortezomib and YM155, for killing KRAS-mutant cancer cells using 3D BEST derived from KRAS-mutant (HCT-116) and WT RAS (HT29) CRC cells. By elucidating the differential potency and selectivity of ADT-007, we aim to accelerate the development of more efficacious and precision treatment strategies for patients with KRAS-mutant CRC. Further, findings from this study will highlight the potential of emerging 3D bioprinted organoid models as an ex vivo assay in preclinical evaluation of target-directed experimental anticancer drugs.

## 2. Materials and Methods

### 2.1. Bioprinting 3D Tumor Models

Using previously established bioprinting protocols [39,40,44], WT RAS and KRAS-mutant 3D BEST constructs were bioprinted with HT29 and HCT-116 cells, respectively. Briefly, BEST constructs were fabricated using a custom bioprinting platform that enabled multiple deposition modes, including layer-by-layer deposition and dynamic bioink mixing approaches. Dynamic bioink composition allowed for modulating collagen density, cell fractions, and other tumor microenvironment parameters, enabling tunable mechanical and biochemical properties. High-throughput bioprinting was achieved through proprietary hardware and software adaptations of epMotion (Eppendorf) robotic fluid handling systems, incorporating custom components including 3D-printed modules and assemblies. These modifications enabled precise control over print speed, real-time dilution, agitation, and temperature regulation throughout the bioprinting process. The integration of passive mixing units facilitated uniform cell distribution for some viscous bioink formulations while maintaining cell viability. The platform’s modular design allows for rapid switching between different bioink compositions and printing parameters, supporting the generation of constructs with spatially defined cellular and matrix distributions that recapitulate key aspects of the native tumor microenvironment.

For all studies, HT29, HCT-116, COLO-205, and DLD-1 cell lines were acquired from ATCC. NCM-460 cells and bespoke culture media were acquired from InCell, Inc. [45]. HT29 is a human WT RAS CRC cell line harboring a BRAF^V600E^ mutation with known sensitivity to proteosome and survivin inhibitors. HCT-116 is a KRAS^G13D^ mutant human CRC cell line. Both cell lines were cultured in Dulbecco’s Modified Eagle Media (DMEM) with 10% fetal bovine serum (FBS), 1% Primosin, and 1% penicillin-streptomycin at 37 °C, 5% CO_2_. Cell viability and counts were assessed using the Countess Automated Cell Counter (Vitrogen) with trypan blue. Cell suspensions with <90% live cells were excluded from bioprinting. Bioink was kept on ice to prevent premature gelation. After printing, BEST constructs were examined for discoloration, bubbles, or other morphological defects using bright field microscopy; only defect-free BEST with uniform cell distribution was utilized for drug screening. Three-dimensional BEST ranged from 300 to 500 µm in thickness. BEST constructs in clear 384-well plates were used for high-content imaging analysis, while those in white 384-well plates were used for metabolic activity measurement with ATP CellTiter-Glo luminescence assay. BEST constructs were allowed to acclimate for 24 h before drug treatment. All experiments were conducted in at least three biological replicates and at least three technical replicates. All observations and readouts were also made in triplicate.

### 2.2. High-Throughput Drug Treatment

Bortezomib (proteasome inhibitor, MW 384.2) and YM155 (survivin inhibitor, MW 443.3) were acquired from Cayman Chemical at 10 mM in DMSO. Gefitinib and GW5074 were obtained from Selleck Chemicals and dissolved in DMSO. ADT-007 (pan-RAS inhibitor, proprietary compound) was provided by Auburn University at 10 mM in DMSO. Each compound was diluted in DMSO and aliquoted at 160 μM to reduce freeze–thaw cycles. Serial dilution and drug administration were performed using a custom-adapted epMotion 5070 (Eppendorf) robotic fluid handling system. The maximum concentration of 1000 nM for each drug was freshly prepared before each experiment from a 160 μM stock solution. After dilution, DMSO in all drug solutions was kept below 2% (*v*/*v*). Drug solutions were transferred to arrays of sterile PCR tubes for automated dispensing. BEST constructs were treated with 11 μL of drug at the following concentrations: 0.03 nM, 0.3 nM, 2 nM, 17 nM, 130 nM, and 1000 nM. Control BEST (0 nM) constructs were treated with 11 μL of 0% FBS/DMEM (control: media only, no drug). Following drug treatment, BEST constructs were incubated under gentle orbital agitation at 37 °C, 5% CO_2_ for 72 h.

### 2.3. High-Content Imaging

A fluorescent dual-stain cocktail was prepared using Hoechst 33342 (10 μg/mL; Thermo Fisher Scientific, cat. no. H3570, Invitrogen, MA, USA) and SYTOX Green (1 μM; Thermo Fisher Scientific, cat. no. S7020) in calcium- and magnesium-free Hank’s Balanced Salt Solution (HBSS; cat. no. 14175095, Gibco, MA, USA). The staining solution was freshly prepared before each experiment and protected from light exposure. Three-dimensional BEST constructs in optically clear 384-well plates (Corning, cat. no. 3712, NY, USA) were stained then incubated at 37 °C, 5% CO_2_ under gentle orbital agitation for 2 h to ensure deep-tissue uniform staining. Automated high-content multi-plane imaging and image processing were performed using a LionHeart Imaging System (Agilent/Biotek, CA, USA.) with specialized custom protocols designed for analyzing 3D BEST. Imaging protocol was optimized to capture autofocus z-stack images of Hoechst 33342 (excitation: 377/50 nm, emission: 447/60 nm) and SYTOX Green (excitation: 469/35 nm, emission: 525/39 nm) stained nuclei covering the entire area of each well at each z-plane, ensuring comprehensive data collection for each BEST construct. Image acquisition parameters, including LED intensity, gain, and integration time, were optimized to maximize signal-to-noise ratio while minimizing phototoxicity and photobleaching. Image analysis was performed using Gen5 imaging software (BioTek Instruments, Gen5 v3.15). Total cells in each BEST construct were approximated by quantifying sum of areas of stained nuclei. Image processing and analysis modules were employed for nuclear segmentation, fluorescence intensity, and sums of object area quantification. Threshold values for Hoechst and SYTOX Green positivity were determined empirically and applied consistently across all analyzed images.

### 2.4. ATP Cell Viability Assay

Cell viability was quantified using the CellTiter-Glo Luminescent Cell Viability Assay (Promega, Madison, WI, USA), with modifications to the manufacturer’s protocol. This assay has been shown to be an excellent indicator for cellular activity in medium and high throughput screening of single cell types for its versatility and sensitivity [46]. The assay exploits the direct correlation between luminescence and cell number over three orders of magnitude, based on the luciferase-catalyzed mono-oxygenation of luciferin in the presence of Mg^2+^, ATP, and molecular oxygen. Thus, the amount of ATP present in the well containing the BEST is directly proportional to the luminescence read from that well, so as the concentration of an active inhibitor increases, luminescence is expected to decrease. After the reagent mixture was added to 3D BEST in white 384-well plates, the plates were gently agitated on an orbital shaker at room temperature for 4 min to facilitate cell lysis, followed by a 20 min static incubation in a dark chamber maintained at room temperature to stabilize the luminescent signal. For monolayer cultures, cells were plated at the concentration required to achieve 80–90% confluent cultures in black 384-well plates, then treated with the indicated compounds for 72 h. An equal volume of CellTiter-Glo was added followed by 10 min incubation protected from light. Luminescence was quantified using a Biotek Synergy HT plate reader. Cell viability was expressed as a percentage relative to untreated control wells. GraphPad Prism software (v10.5.0) was used to determine IC_50_ values and generate dose–response curves using logistic regression.

### 2.5. Apoptosis Assay

Cell lines were plated in 6-well plates and allowed to grow to 60% confluence. Cells were then incubated with vehicle (0.1% DMSO) or ADT-007 at the indicated concentrations for 72 h before washing, collection by trypsinization, and staining with propidium iodide/Annexin V according to the kit manufacturer’s protocol (BD Pharmingen, San Diego, CA, USA). Cells were analyzed via flow cytometry using a BD-FACS Canto II (Becton-Dickinson, San Jose, CA, USA). The percentage of apoptotic cells was calculated using DIVA software version 6.1.3 (Becton-Dickinson).

### 2.6. Generative AI Disclosure

The graphical abstract was created with the assistance of ChatGPT (OpenAI o4-mini), which was used to generate the design elements and layout. No additional AI tools were used for data analysis or interpretation.

## 3. Results

### 3.1. RAS Selective Inhibition of CRC Cell Growth by ADT-007

**Figure 1:**
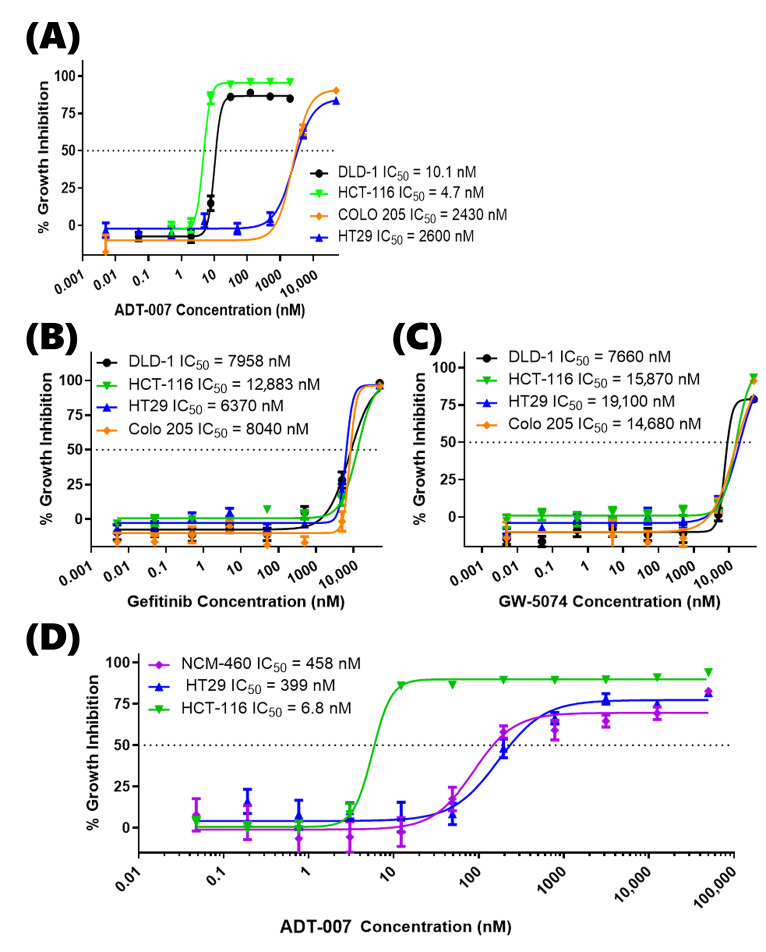
Growth inhibitory potency and RAS selectivity of ADT-007 in standard 2D monolayer cultures of RAS mutant and WT CRC cell lines. (**A**) Dose-dependent growth inhibition of KRAS-mutant (DLD-1, HCT-116) versus RAS WT (HT29, COLO-205) cell lines by ADT-007 as determined by cell viability assays following 72 h of treatment (Promega CellTiter-Glo assay). (**B**) Dose-dependent growth inhibition of KRAS-mutant (DLD-1, HCT-116) versus RAS WT (HT29, COLO-205) cell lines by EGFR inhibitor, Gefitinib. (**C**) Dose-dependent growth inhibition of KRAS-mutant (DLD-1, HCT-116) versus RAS WT (HT29, COLO-205) cell lines by RAF1 inhibitor, GW-5074. (**D**) Dose-dependent growth inhibition of KRAS-mutant (HCT-116) versus RAS WT (HT29) and normal colon mucosa (NCM-460) cells. Data points represent mean ± SEM of four replicates, each from *n* = 2 independent experiments.

Potency and selectivity of ADT-007 to inhibit CRC cell growth were initially determined using two KRAS-mutant and two RAS WT human CRC cell lines grown in monolayer culture following 3 days of treatment and measured using the CellTiter-Glo luminescence assay. We confirmed expected KRAS mutational status via genotype and literature-reported expression levels in all cell lines, and proceeded to measure growth IC_50_ values by CellTiter-Glo in 2D monolayers. The growth IC_50_ values of ADT-007 for the G13D mutant KRAS DLD-1 and HCT-116 lines were 10.1 and 4.7 nM, respectively (Figure 1A). By comparison, the growth IC_50_ values of ADT-007 for the WT RAS mutant BRAF HT29 and COLO 205 lines were 2600 and 2430 nM, respectively. We confirmed the reported dependence of these cell lines on the EGFR and MAPK pathway. We tested the growth inhibitory activity in the same cell lines with Gefitinib, an inhibitor of the EGF receptor immediately upstream of RAS (Figure 1B) or with GW-5074, an inhibitor of the RAF1 kinase immediately downstream of RAS (Figure 1C). Other experiments revealed that normal colon mucosa cells (NCM-460) were as insensitive to ADT-007 treatment as RAS WT HT29 cells (Figure 1D).

### 3.2. RAS Selective Apoptosis Induction by ADT-007

**Figure 2:**
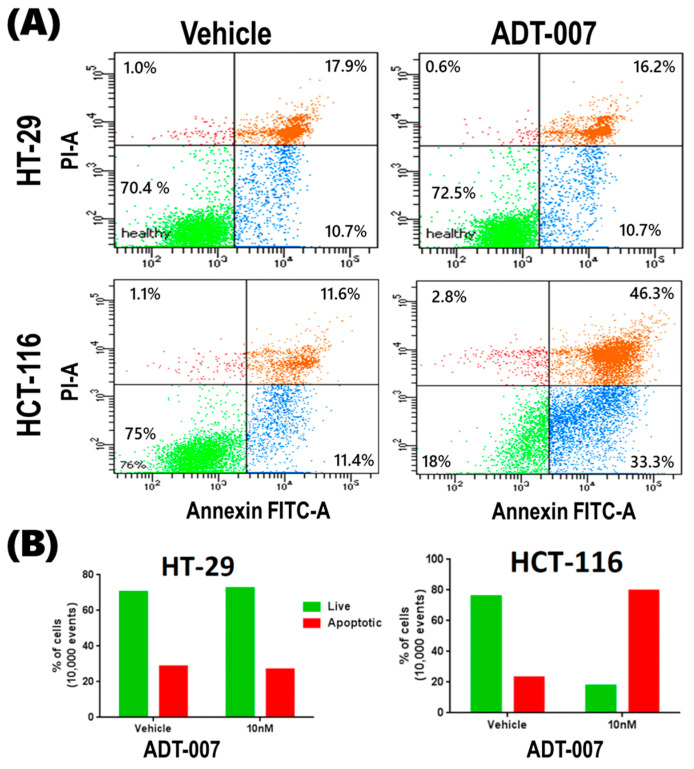
RAS selective apoptosis induction by ADT-007. (**A**) ADT-007 induces apoptosis of KRAS-mutant HCT-116 cells, but not RAS WT HT29 cells, as measured by flow cytometry based on Annexin V levels. (**B**) Flow cytometry measurements of Annexin V and propidium iodide levels. Cells were plated in 6-well dishes and treated with vehicle (0.1% DMSO) or ADT-007 at the indicated concentration for 72 h before staining with propidium iodide and Annexin V and analysis by flow cytometry. Results reflect 10,000 events recorded in a single experiment.

Additional experiments revealed that ADT-007 (72 h treatment) significantly induced apoptosis of KRAS-mutant HCT-116 cells (Figure 2A), but not RAS WT HT29 cells (Figure 2B), measured by flow cytometry, using Annexin V as a biochemical marker of apoptosis.

### 3.3. Differential Response in KRAS-Mutant CRC BEST

**Figure 3:**
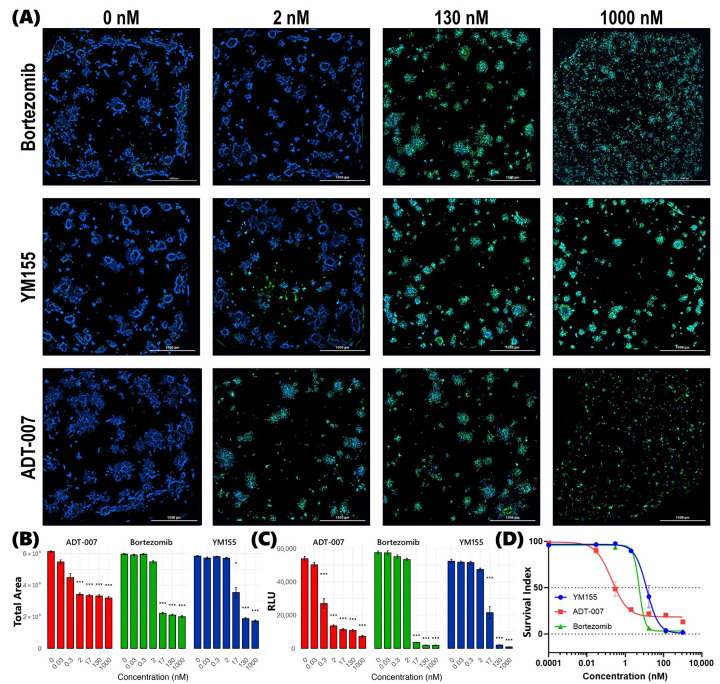
Superior potency of ADT-007 benchmarked against mechanistically distinct bortezomib and YM155 to inhibit the growth of KRAS-mutant CRC BEST. (**A**) Representative high-content fluorescence micrographs of nuclear-stained BEST generated with KRAS-mutant HCT-116 CRC cells. The panels show the effect of treating BEST with increasing concentrations (left to right) from 0 nM (vehicle) to 1000 nM of bortezomib (**top**), YM155 (**middle**), and ADT-007 (**bottom**). All images were acquired for multiple z-planes and resolved into single image using a LionHeart imaging system with a 4X objective after treating BEST for 72 h. Blue: Hoechst 33342 (total nuclei); Green: SYTOX Green (dead cells). Scale bar = 1000 µm. (**B**) Dose–response relationship between drug concentration and total area occupied by cells derived from high-content imaging analysis. As the drug concentration increases, total cell area decreases for all three drugs. Drug potency was calculated based on the reduction in area of Hoechst-positive nuclei and normalized to independent untreated controls. Data points represent mean ± SEM from *n* = 3 independent experiments, each performed in triplicate. Statistical analysis: * *p* < 0.05, *** *p* < 0.001 in pairwise *t*-test against control for each drug concentration. (**C**) Confirmatory dose response from CellTiter-Glo luminescence assay expressed as relative luminescence units (RLUs) was obtained in parallel with high-content imaging studies under identical treatment conditions. Data representation and statistical analysis as described above. (**D**) Dose–response curves generated from CellTiter-Glo luminescence data using nonlinear regression in GraphPad Prism. Curves were fitted using four-parameter logistic regression. IC_50_ values: ADT-007 = 0.3 nM; bortezomib = 5.8 nM; YM155 = 5.4 nM. *p* < 0.01 for ADT-007 vs. bortezomib and YM155.

In prior preclinical studies by other investigators, bortezomib [16,17] and YM155 [23,24] were reported to inhibit the growth of CRC cell lines, including KRAS-mutant HCT-116 and WT RAS HT29. While both drugs were found to be potent inhibitors, the magnitude of response varied among studies. For instance, studies conducted with HT29 by Pitts et al. [47] reported an IC_50_ of 500 nM for bortezomib, while Suzuki et al. [48] reported an IC_50_ of 13 nM. Furthermore, since many such studies have been conducted using conventional 2D monolayer cell cultures, we sought to determine growth inhibitory activity for both agents using 3D BEST, that we hypothesize will be more predictive of anticancer activity, and compare the response with ADT-007, a novel pan-RAS inhibitor. To evaluate anticancer efficacy in our study, a cell population of less than 70% of that in control BEST at endpoint was used as a proxy for the RECIST criteria of a 30% reduction in tumor burden. This measure reflects the combined effects of growth inhibition and cell death in treated BEST compared to continued growth observed in control BEST. In KRAS-mutant BEST, generated with HCT-116 cells, bortezomib was found to be more effective than YM155, which was consistent with efficacy profiles reported by other investigators and compiled in the NCI-60 Growth Inhibition Database [49,50]. Interestingly, for these BEST constructs, ADT-007 was appreciably more potent than bortezomib and YM155. High-content image analysis of post-treatment BEST stained with Hoechst 33342 and SYTOX (Figure 3A,B and Figure S1) confirmed that ADT-007 achieved a >30% reduction in tumor burden at lower concentrations (2 nM) when benchmarked against both bortezomib (17 nM) and YM155 (17 nM). Metabolic activity, as measured with an ATP CellTiter-Glo luminescence assay, was suppressed with lower concentrations of ADT-007 than either bortezomib or YM155, which is consistent with results from high-content imaging. IC_50_ values for ADT-007, bortezomib, and YM155, derived from metabolic activity, were 0.3 nM, 5.8 nM, and 5.4 nM, respectively (Figure 3C,D). As we previously reported, ADT-007 can induce mitotic arrest in short term cultures, mimicking an incomplete efficacy. However, it was previously shown that the residual cells may remain metabolically active for a time, but are incapable of continued proliferation as determined by a clonogenic assay [35].

### 3.4. Differential Response in WT RAS CRC BEST

**Figure 4:**
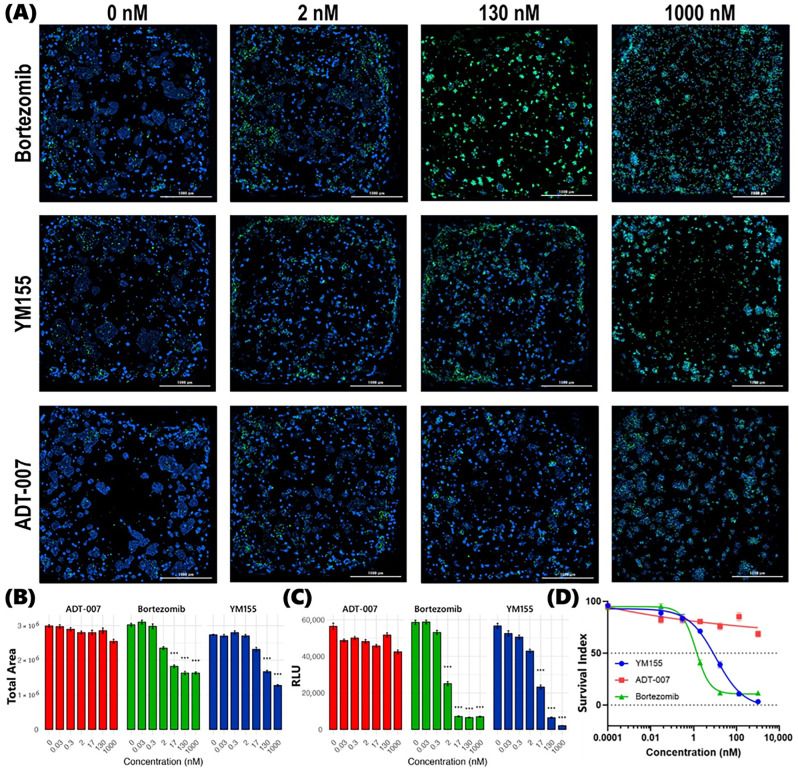
Superior selectivity of ADT-007 benchmarked against mechanistically distinct bortezomib and YM155 to inhibit the growth of WT RAS CRC BEST. (**A**) Representative high-content fluorescence micrographs of nuclear-stained BEST generated with WT RAS HT29 CRC cells. The panels show the effect of treating with increasing concentrations (left to right) from 0 nM (vehicle) to 1000 nM of bortezomib (**top**), YM155 (**middle**), and ADT-007 (**bottom**). Images acquired using a LionHeart imaging system with a 4X objective after BEST were treated for 72 h. Blue: Hoechst 33342 (total nuclei); Green: SYTOX Green (dead cells). Scale bar = 1000 µm. (**B**) Dose–response relationship between drug concentration and total cell area (Hoescht stain). As drug concentration increases for bortezomib and YM155, blue pixel area decreases, indicating an inhibitory effect on cell proliferation. This effect is not observed with ADT-007, as blue area remains nearly constant along the concentration gradient. Drug efficacy was calculated based on reduction in area of Hoechst-positive nuclei and normalized to independent untreated controls. Data points represent mean ± SEM from *n* = 3 independent experiments, each performed in triplicate. Statistical analysis: *** *p* < 0.001 in pairwise *t*-test against control for each drug concentration. (**C**) Confirmatory dose response from CellTiter-Glo luminescence assay expressed as relative luminescence units (RLUs) was obtained in parallel with high-content imaging studies under identical treatment conditions. Data representation and statistical analysis as in (**B**). (**D**) Dose response curves generated from CellTiter-Glo luminescence data using nonlinear regression in GraphPad Prism. Curves were fitted using four-parameter logistic regression. IC_50_ values: ADT-007 > 1000 nM; bortezomib = 1.6 nM; YM155 = 7.2 nM. *p* < 0.01 for ADT-007 vs. bortezomib and YM155.

In WT RAS BEST generated with HT29 cells, bortezomib was found to be more effective than YM155, which was consistent with differential efficacy profiles reported by other investigators and compiled in the NCI-60 Growth Inhibition Database [49,50]. While both bortezomib and YM155 showed potency at low concentrations in WT RAS BEST, ADT-007 was completely inactive, which reflects its unique selectivity for RAS mutant cancer cells. This was evident in both high content image analysis of post-treatment BEST stained with Hoechst 33342 and SYTOX (Figure 4A,B and Figure S2) and ATP CellTiter-Glo luminescence assays (Figure 4C,D). The IC_50_ values for bortezomib and YM155 were 1.6 nM and 7.2 nM, respectively, while the IC_50_ for ADT-007 was greater than 1000 nM.

### 3.5. Summary of Differential Response in CRC BEST

A clear dose-dependent response was observed for ADT-007, bortezomib, and YM155 to inhibit the growth of KRAS-mutant CRC BEST. High-content fluorescence images, captured at multiple z-planes of post-treatment nuclear-stained BEST, show an increasing proportion of dead cells (SYTOX Green positive) relative to the total number of cells (Hoechst 33342 positive) in tandem with increasing concentrations for each drug. This trend was consistent across all three drugs, albeit with varying degrees of potency. While a similar dose-dependent response was observed in WT RAS BEST for bortezomib and YM155, sensitivity to ADT-007 was at least one-hundred-fold lower in these BEST compared to KRAS-mutant HCT-116 BEST. Setting quantitative image analysis aside, qualitative visual comparison of cell death (SYTOX Green positive) captured in Figure 3A (KRAS-mutant HCT-116 BEST) with that in Figure 4A (WT RAS HT29 BEST) clearly indicates only a modest effect of ADT-007 in WT RAS BEST, even at a 130 nM dose, vis-à-vis high potency at only 2 nM in KRAS-mutant BEST. These observations were quantified and further confirmed by Gen5 image processing algorithms. Specifically, tumor burden decreased with increasing drug concentrations for bortezomib and YM155 in both WT and KRAS-mutant BEST. However, this trend was only observed for ADT-007 in KRAS-mutant BEST, resulting in IC_50_ values for ADT-007 of >1000 nM in WT RAS BEST (Figure 4D) compared to 0.3 nM in KRAS-mutant BEST (Figure 3D). These results are consistent with previous reports that ADT-007 selectively inhibits mutant RAS by blocking GTP activation of RAS-effector interactions [35]. Similar dose-dependent response profiles were observed in confirmatory CellTiter-Glo luminescence assays. While a significant decrease in luminescence was recorded along the concentration gradient for bortezomib and YM155 in both WT RAS (Figure 4C) and KRAS-mutant BEST (Figure 3C), such a trend was limited to KRAS-mutant BEST for ADT-007, resulting in IC_50_ values that align well with those derived from high-content imaging for all three drugs in both WT RAS and KRAS-mutant BEST. These results suggest that ADT-007 exhibited superior efficacy in KRAS-mutant HCT-116 BEST when benchmarked against both bortezomib and YM155, as evidenced by lower IC_50_ values in both the high-content imaging cell viability analyses and the confirmatory metabolic activity assay. This enhanced potency of ADT-007 in KRAS^G13D^-mutant HCT-116 CRC BEST aligns with its mechanism of action, targeting constitutively activated RAS to block downstream signaling that drives proliferation and survival of these cells. The differential, selective response observed for the targeted therapy (ADT-007) benchmarked against the response to both bortezomib and YM155 highlights the need for further development and of pan-RAS inhibitors such as ADT-007 in treating RAS-mutant CRC.

## 4. Discussion

We recently characterized the ultra-high potency and unique selectivity of ADT-007 to kill cancer cell lines with mutant RAS [35]. Briefly, ADT-007 showed potent allele-specific activity across a broad panel of RAS-mutant cancer cell lines including HCT116 (KRAS^G13D^), MIA PaCa-2 (KRAS^G12C^), PANC-1 (KRAS^G12D^), SW480 (KRAS^G12V^), and A549 (KRAS^G12S^), while displaying little to no activity in RAS wild-type, BRAF-mutant lines such as HT29 and BxPC-3. We also developed an orally bioavailable prodrug of ADT-007, coded as ADT-1004, that shows strong and robust antitumor activity in multiple models of pancreatic cancer, including clinically relevant patient-derived xenograft models [37]. Despite its broad growth inhibitory activity, ADT-007 displayed exquisite target specificity, as cancer cells with WT RAS but downstream BRAF mutations, as well as cells from normal tissue, were essentially insensitive to ADT-007. The specificity of ADT-007 to kill cancer cells with mutant RAS was attributed to its ability to block GTP activation of RAS and high levels of activated RAS in cancer cells harboring RAS mutations and their dependence on RAS signaling for proliferation and survival, commonly referred to as “RAS addiction”. The requirement of activated RAS for the growth inhibitory activity of ADT-007 was evidenced from experiments shown here, where high potency and selectivity to kill CRC cells with mutant KRAS was associated with high levels of activated RAS [35]. We also showed that ADT-007 treatment of such cells decreased GTP-RAS levels and inhibited MAPK signaling [35]. The RAS-selectivity of ADT-007 was not replicated by either upstream or downstream inhibitors of RAS signaling in these cell models. As previously reported and confirmed here, ADT-007 selectively induces apoptosis of KRAS-mutant cancer cells, which may be a key advantage over mutant-specific KRAS and other pan-KRAS or pan-RAS inhibitors approved or in development by circumventing resistance [35].

While initial experiments with ADT-007 were performed in 2D monolayer cultures, there is considerable evidence that 3D tissue cultures are superior to 2D monolayer cell cultures for modeling malignant disease [41,42,43]. For example, other investigators have demonstrated greater physiological relevance of 3D cultures to in vivo tumorigenicity, metabolic activity, and protein expression compared to that observed in 2D cultures [51,52]. Thus, in this study we developed and utilized simple 3D BEST as proxies of WT RAS and KRAS^G13D^-mutant CRC tumors to further study the potency and selectivity of ADT-007 in more predictive preclinical models. Compared to 2D monolayer cultures, the 3D microarchitecture of BEST can also better replicate potential variations in drug penetration, uptake, cellular response in different regions, and multidimensional influences of drug exposure that would not be readily evident in numerical metabolic activity assays of monolayer cell cultures. For instance, beyond assessing the inhibitory or cytotoxic potential of drugs, the approach in this study that combines 3D BEST with multiplane high-content imaging and a confirmatory conventional metabolic activity ATP assay also enables the elucidation of conformational and morphological changes in the microarchitecture of the 3D tumor mimics. Notably, the disruption of characteristic 3D clustering of CRC cells, as apparent in multi-plane high-content micrographs of post-treatment BEST, was evident at concentrations appreciably below growth IC_50_ values in 2D monolayer cultures. This high-dimensional data could be utilized as training and testing data for emerging innovative in silico drug discovery systems. While this approach may sacrifice the speed associated with conventional 2D cell viability assays, the depth and relevance of data obtained could more than compensate for the trade-off. Importantly, our observations from high-content imaging were corroborated by confirmatory metabolic ATP assays, which reported similar dose-dependent trends. This concordance between orthogonal assays strengthens the validity of our findings and underscores the robustness of the 3D BEST model for drug screening. Moreover, the agreement between these distinct methodologies suggests that the observed drug effects are not artifacts of a particular assay.

While our 3D BEST model offers advantages over conventional monolayers, it is important to note its limitations. The BEST constructs utilized in our study lack key components of the tumor microenvironment, including immune cells, stromal cells, and a complex extracellular matrix [53]. This simplified microenvironment of our BEST constructs reduces time, complexity, and resources, but it should be noted that this comes at the cost of failing to recapitulate the complex interactions between tumor cells and their surroundings. Additionally, the absence of vasculature in BEST may affect drug distribution in ways that differ from in vivo tumors, potentially impacting our assessment of drug efficacy. It would also be naïve to expect that these findings could be directly applicable in vivo, particularly in clinical trials and in the clinic. Factors such as drug metabolism, clearance, and potential off-target effects in other tissues cannot be fully assessed in our simplified 3D BEST. Despite these limitations, we believe our BEST constructs provide valuable preclinical insights and demonstrate the potential of high-content multidimensional assays as an effective intermediate step toward more complex patient-derived models. Our study highlights the value of this approach in assessing differential potency and selectivity of ADT-007 in KRAS^G13D^-mutant versus WT RAS CRC BEST benchmarked against well-investigated drugs such as bortezomib and YM155. This enhanced performance in KRAS^G13D^-mutant BEST, coupled with its reduced cytotoxic effect on WT RAS CRC BEST, suggests that ADT-007 may offer a more selective approach to targeting cancer cells with KRAS^G13D^-mutational profiles. However, it will be crucial to validate these findings in more complex systems, including patient-derived BEST constructs, which incorporate both tumor and stromal cells from the patient’s own tumor, and eventually in carefully designed clinical trials.

## 5. Conclusions

Looking ahead, we recognize the need for further investigation to bridge gaps between these preclinical findings and potential clinical utility. Our planned studies, using patient tissue and patient-derived BEST that incorporate both tumor and stromal cells from the patient’s own tumor, aim to provide more clinically relevant insights into the efficacy and target specificity of ADT-007, and potential advantages over other RAS inhibitors approved or in development. Additionally, investigation into potential resistance mechanisms and combination therapies could further enhance the translational potential of ADT-007 and other pan-RAS targeted strategies. In conclusion, while acknowledging the limitations of our current model, this study represents a significant step forward in the preclinical evaluation of targeted therapies for KRAS-mutant CRC. By demonstrating the potential of both ADT-007 and the 3D BEST model, we lay the groundwork for future investigations that could ultimately lead to more effective and personalized treatment strategies for patients with KRAS-mutant CRC and other RAS-driven cancers.

## Data Availability

Additional data available upon request.

## Supplementary Materials

The following supporting information can be downloaded at https://www.mdpi.com/article/10.3390/cancers17182958/s1, Figure S1. Dose–response relationship between drug concentration and proportion of dead cells in HCT-116 BEST as determined from high-content imaging analysis of SYTOX-positive cells; Figure S2. Dose–response relationship between drug concentration and proportion of dead cells in HT29 BEST as determined from high-content imaging analysis of SYTOX-positive cells.

## Abbreviations

The following abbreviations are used in this manuscript:

BEST	Bioprinted ex vivo Slice Tissue
CRC	Colorectal cancer
WT	Wild-type

## References

[1] Bray F., Laversanne M., Sung H., Ferlay J., Siegel R.L., Soerjomataram I., Jemal A. (2024). Global cancer statistics 2022: GLOBOCAN estimates of incidence and mortality worldwide for 36 cancers in 185 countries. CA. Cancer J. Clin..

[2] Deo S.V.S., Kumar S., Bhoriwal S., Shukla N.K., Sharma A., Thulkar S., Das P., Bhagat P., Dhall K., Pathy S. (2021). Colorectal Cancers in Low- and Middle-Income Countries—Demographic Pattern and Clinical Profile of 970 Patients Treated at a Tertiary Care Cancer Center in India. JCO Glob. Oncol..

[3] Abdelgadir O., Kuo Y.-F., Khan M.F., Okorodudu A.O., Cheng Y.-W., Dong J. (2025). Mortality Outcome Associated with Specific KRAS, NRAS, and BRAF Hot-Spot Mutations in Metastatic Colorectal Cancer Patients: A Retrospective Cohort Study. Diagnostics.

[4] Zhu G., Pei L., Xia H., Tang Q., Bi F. (2021). Role of oncogenic KRAS in the prognosis, diagnosis and treatment of colorectal cancer. Mol. Cancer.

[5] Simanshu D.K., Nissley D.V., McCormick F. (2017). RAS Proteins and Their Regulators in Human Disease. Cell.

[6] Prior I.A., Hood F.E., Hartley J.L. (2020). The frequency of ras mutations in cancer. Cancer Res..

[7] Stephen A.G., Esposito D., Bagni R.K., McCormick F. (2014). Dragging ras back in the ring. Cancer Cell.

[8] Charette N., Vandeputte C., Stärkel P. (2014). Ras in digestive oncology: From molecular biology to clinical implications. Curr. Opin. Oncol..

[9] Piazza G.A., Chandrasekaran P., Maxuitenko Y.Y., Budhwani K.I. (2024). Assessment of KRASG12C inhibitors for colorectal cancer. Front. Oncol..

[10] Porru M., Pompili L., Caruso C., Biroccio A., Leonetti C. (2018). Targeting kras in metastatic colorectal cancer: Current strategies and emerging opportunities. J. Exp. Clin. Cancer Res..

[11] Bteich F., Mohammadi M., Li T., Bhat M.A., Sofianidi A., Wei N., Kuang C. (2023). Targeting KRAS in Colorectal Cancer: A Bench to Bedside Review. Int. J. Mol. Sci..

[12] Cox A.D., Fesik S.W., Kimmelman A.C., Luo J., Der C.J. (2014). Drugging the undruggable RAS: Mission Possible?. Nat. Rev. Drug Discov..

[13] Papke B., Der C.J. (2017). Drugging RAS: Know the enemy. Science.

[14] Moore A.R., Rosenberg S.C., McCormick F., Malek S. (2020). RAS-targeted therapies: Is the undruggable drugged?. Nat. Rev. Drug Discov..

[15] Manasanch E.E., Orlowski R.Z. (2017). Proteasome inhibitors in cancer therapy. Nat. Rev. Clin. Oncol..

[16] Mackay H., Hedley D., Major P., Townsley C., Mackenzie M., Vincent M., Degendorfer P., Tsao M.-S., Nicklee T., Birle D. (2005). A Phase II Trial with Pharmacodynamic Endpoints of the Proteasome Inhibitor Bortezomib in Patients with Metastatic Colorectal Cancer. Clin. Cancer Res..

[17] Cusack J.C., Liu R., Houston M., Abendroth K., Elliott P.J., Adams J., Baldwin A.S. (2001). Enhanced chemosensitivity to CPT-11 with proteasome inhibitor PS-341: Implications for systemic nuclear factor-κB inhibition. Cancer Res..

[18] Altieri D.C. (2008). Survivin, cancer networks and pathway-directed drug discovery. Nat. Rev. Cancer.

[19] Krieg A., Werner T.A., Verde P.E., Stoecklein N.H., Knoefel W.T., Srinivasula S.M. (2013). Prognostic and Clinicopathological Significance of Survivin in Colorectal Cancer: A Meta-Analysis. PLoS ONE.

[20] Rauch A., Carlstedt A., Emmerich C., Mustafa A.-H.M., Göder A., Knauer S.K., Linnebacher M., Heinzel T., Krämer O.H. (2018). Survivin antagonizes chemotherapy-induced cell death of colorectal cancer cells. Oncotarget.

[21] Lee M.-R., Ji S.-Y., Mia-Jan K., Cho M.-Y. (2015). Chemoresistance of CD133+ colon cancer may be related with increased survivin expression. Biochem. Biophys. Res. Commun..

[22] Kim S.T., Sohn I., Do I.-G., Jang J., Kim S.H., Jung I.H., Park J.O., Park Y.S., Talasaz A., Lee J. (2014). Transcriptome analysis of CD133-positive stem cells and prognostic value of survivin in colorectal cancer. Cancer Genom. Proteom..

[23] Rauch A., Hennig D., Schäfer C., Wirth M., Marx C., Heinzel T., Schneider G., Krämer O.H. (2014). Survivin and YM155: How faithful is the liaison?. Biochim. Biophys. Acta-Rev. Cancer.

[24] Xiao M., Li W. (2015). Recent Advances on Small-Molecule Survivin Inhibitors. Curr. Med. Chem..

[25] Fukuda S., Pelus L.M. (2006). Survivin, a cancer target with an emerging role in normal adult tissues. Mol. Cancer Ther..

[26] Nakahara T., Takeuchi M., Kinoyama I., Minematsu T., Shirasuna K., Matsuhisa A., Kita A., Tominaga F., Yamanaka K., Kudoh M. (2007). YM155, a novel small-molecule survivin suppressant, induces regression of established human hormone-refractory prostate tumor xenografts. Cancer Res..

[27] Hong D.S., Fakih M.G., Strickler J.H., Desai J., Durm G.A., Shapiro G.I., Falchook G.S., Price T.J., Sacher A., Denlinger C.S. (2020). KRAS G12C Inhibition with Sotorasib in Advanced Solid Tumors. N. Engl. J. Med..

[28] Jänne P., Rybkin I., Spira A., Riely G., Papadopoulos K., Sabari J., Johnson M., Heist R., Bazhenova L., Barve M. (2020). KRYSTAL-1: Activity and Safety of Adagrasib (MRTX849) in Advanced/Metastatic Non–Small-Cell Lung Cancer (NSCLC) Harboring KRAS G12C Mutation. Eur. J. Cancer.

[29] Vasan N., Boyer J.L., Herbst R.S. (2014). A RAS Renaissance: Emerging Targeted Therapies for KRAS-Mutated Non–Small Cell Lung Cancer. Clin. Cancer Res..

[30] Ryan M.B., de la Cruz F.F., Phat S., Myers D.T., Wong E., Shahzade H.A., Hong C.B., Corcoran R.B. (2020). Vertical pathway inhibition overcomes adaptive feedback resistance to KrasG12C inhibition. Clin. Cancer Res..

[31] McCormick F. (2015). KRAS as a therapeutic target. Clin. Cancer Res..

[32] Herrmann C., Block C., Geisen C., Haas K., Weber C., Winde G., Möröy T., Müller O. (1998). Sulindac sulfide inhibits Ras signaling. Oncogene.

[33] Sattler M., Mohanty A., Kulkarni P., Salgia R. (2023). Precision oncology provides opportunities for targeting KRAS-inhibitor resistance. Trends Cancer.

[34] Shima F., Yoshikawa Y., Ye M., Araki M., Matsumoto S., Liao J., Hu L., Sugimoto T., Ijiri Y., Takeda A. (2013). In silico discovery of small-molecule Ras inhibitors that display antitumor activity by blocking the Ras-effector interaction. Proc. Natl. Acad. Sci. USA.

[35] Foote J.B., Mattox T.E., Keeton A.B., Chen X., Smith F.T., Berry K., Holmes T.W., Wang J., Huang C.-H., Ward A. (2024). A Pan-RAS Inhibitor with a Unique Mechanism of Action Blocks Tumor Growth and Induces Antitumor Immunity in Gastrointestinal Cancer. Cancer Res..

[36] Jiang J., Jiang L., Maldonato B.J., Wang Y., Holderfield M., Aronchik I., Winters I.P., Salman Z., Blaj C., Menard M. (2024). Translational and Therapeutic Evaluation of RAS-GTP Inhibition by RMC-6236 in RAS-Driven Cancers. Cancer Discov..

[37] Bandi D.S.R., Nagaraju G.P., Sarvesh S., Carstens J.L., Foote J.B., Graff E.C., Fang Y.-H.D., Keeton A.B., Chen X., Valiyaveettil J. (2025). ADT-1004: A first-in-class, oral pan-RAS inhibitor with robust antitumor activity in preclinical models of pancreatic ductal adenocarcinoma. Mol. Cancer.

[38] Budhwani K.I., Patel Z.H., Guenter R.E., Charania A.A. (2022). A hitchhiker’s guide to cancer models. Trends Biotechnol..

[39] Bollenbecker S., Patel Z., Punjani Z., Charania A., Patel H., Abbott A., Kunkle K., Sewell-Loftin M.K., Grossman G., Budhwani K. (2023). Predictive efficacy biomarker for chemotherapy agents against triple-negative breast cancer bioprinted organoid tumors (BOTs) using solid tumor biopsy-on-a-chip. Cancer Res..

[40] Patel Z.H., Charania A.A., Punjani Z., Patel H.K., Sewell-Loftin M.K., Saleh M.N., Budhwani K.I. (2022). Evaluating anticancer agents on 3D bioprinted organoid tumors (BOT) to reduce cost and accelerate therapeutic discovery. J. Clin. Oncol..

[41] Ghajar C.M., Bissell M.J. (2010). Tumor Engineering: The Other Face of Tissue Engineering. Tissue Eng. Part A.

[42] Lee G.Y., A Kenny P., Lee E.H., Bissell M.J. (2007). Three-dimensional culture models of normal and malignant breast epithelial cells. Nat. Methods.

[43] Kawai S., Shibuya K., Yamazaki M., Fujii E., Nakano K., Suzuki M. (2020). Three-dimensional culture models mimic colon cancer heterogeneity induced by different microenvironments. Sci. Rep..

[44] Zuaiter D., Ahirwar P., Pokal A.G., Patel Z.H., Charania A.A., Crawford C.L., Sewell-Loftin M.K., Tsung A., Kim A., Budhwani K.I. (2024). Characterizing differential efficacy and phenotypic response to proteasome and survivin inhibitors in colorectal cancers using a high throughput organoid assay. J. Clin. Oncol..

[45] Moyer M.P., Manzano L.A., Merriman R.L., Stauffer J.S., Tanzer L.R. (1996). NCM460 a Normal Human Colon Mucosal Epithelial Cell Line. Vitr. Cell. Dev. Biol.-Anim..

[46] Zimmermann S., Gretzinger S., Scheeder C., Schwab M., Oelmeier S.A., Osberghaus A., Gottwald E., Hubbuch J. (2016). High-throughput cell quantification assays for use in cell purification development—Enabling technologies for cell production. Biotechnol. J..

[47] Pitts T.M., Morrow M., Kaufman S.A., Tentler J.J., Eckhardt S.G. (2009). Vorinostat and bortezomib exert synergistic antiproliferative and proapoptotic effects in colon cancer cell models. Mol. Cancer Ther..

[48] Suzuki E., Demo S., Deu E., Keats J., Arastu-Kapur S., Bergsagel P.L., Bennett M.K., Kirk C.J. (2011). Molecular mechanisms of bortezomib resistant adenocarcinoma cells. PLoS ONE.

[49] Shoemaker R.H. (2006). The NCI60 human tumour cell line anticancer drug screen. Nat. Rev. Cancer.

[50] NCI (2012). NCI-60 Growth Inhibition Data. NCI DTP Data.

[51] Santini M.T., Rainaldi G., Romano R., Ferrante A., Clemente S., Motta A., Indovina P.L. (2004). MG-63 human osteosarcoma cells grown in monolayer and as three-dimensional tumor spheroids present a different metabolic profile: A 1H NMR study. FEBS Lett..

[52] Pampaloni F., Reynaud E.G., Stelzer E.H.K. (2007). The third dimension bridges the gap between cell culture and live tissue. Nat. Rev. Mol. Cell Biol..

[53] Boykin L.A., Jayashankar S., Budhwani K.K.K., Budhwani B.M.K., Samal D., Crawford C.L., Tsung A., Budhwani K.I. (2025). Protocol for spatial characterization of ECM collagen-GAG in colorectal cancer tumor microenvironment. arXiv.

