# Supplementary material for: Evaluation of a Novel Pan-RAS Inhibitor in 3D Bioprinted Tumor Models
Source: Cancers (Basel). 2025 Sep 10;17(18):2958. doi: 10.3390/cancers17182958 (PMC12468595; doi:10.3390/cancers17182958)
Supplement: Supplementary file 1 [file cancers-17-02958-s001.zip › cancers-3825429-supplementary.pdf]

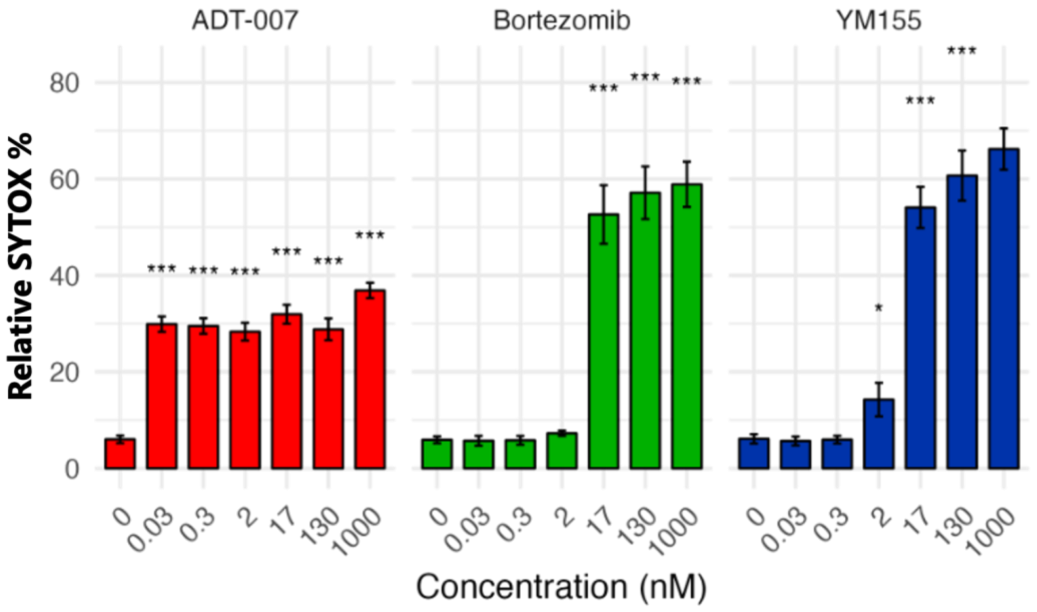

Figure S1. Dose-response relationship between drug concentration and proportion of dead cells in HCT-116 BEST as determined from high-content imaging analysis of SYTOX-positive cells. As drug concentration increases, the proportion of dead cells increases for all three drugs, with distinct potency profiles observed. Proportion of dead cells was quantified based on SYTOX-positive nuclei relative to total Hoechst-positive nuclei and normalized to independent untreated controls. Data points represent mean ± SEM from n = 3 independent experiments, each performed in triplicate. Statistical analysis: *$p < 0.05$, ***$p < 0.001$ in pairwise t-test against control for each drug concentration.

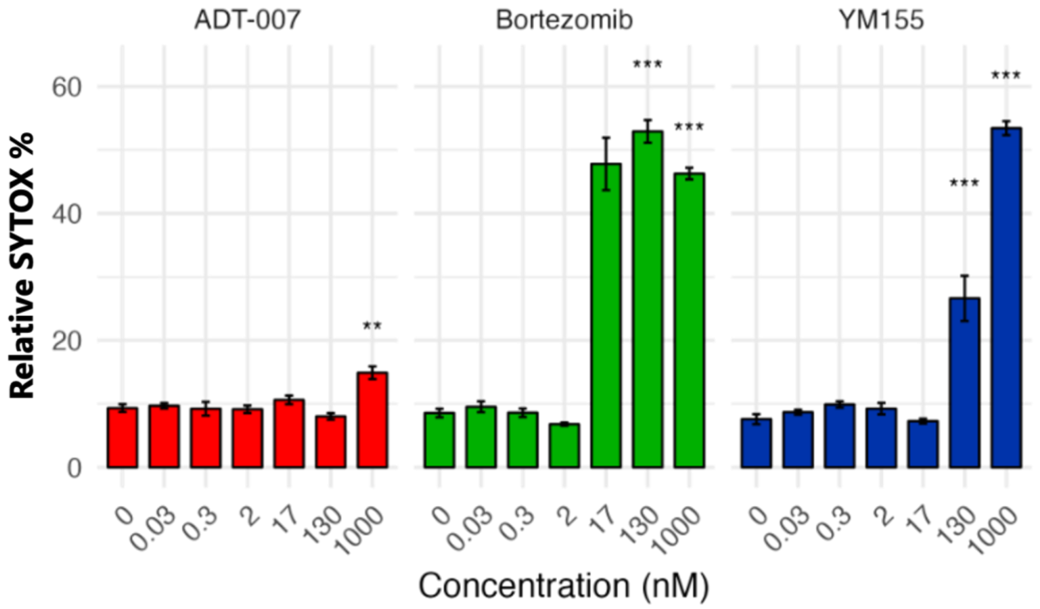

Figure S2. Dose-response relationship between drug concentration and proportion of dead cells in HT29 BEST as determined from high-content imaging analysis of SYTOX-positive cells. As drug concentration increases, the proportion of dead cells shows minimal increase for ADT-007 but significant increases for bortezomib and YM155 at higher concentrations, demonstrating distinct potency and selectivity profiles. Proportion of dead cells was quantified based on SYTOX-positive nuclei relative to total Hoechst-positive nuclei and normalized to independent untreated controls. Data points represent mean ± SEM from n = 3 independent experiments, each performed in triplicate. Statistical analysis: **$p < 0.01$, ***$p < 0.001$ in pairwise t-test against control for each drug concentration.
